# Efficient Removal of Chromium(VI) Anionic Species and Dye Anions from Water Using MOF-808 Materials Synthesized with the Assistance of Formic Acid

**DOI:** 10.3390/nano11061398

**Published:** 2021-05-25

**Authors:** Khoa D. Nguyen, Phuoc H. Ho, Phuong D. Vu, Thuyet L. D. Pham, Philippe Trens, Francesco Di Renzo, Nam T. S. Phan, Ha V. Le

**Affiliations:** 1 Department of Chemical Engineering, Ho Chi Minh University of Technology, 268 Ly Thuong Kiet Street, District 10, Ho Chi Minh City 740010, Vietnam; phuong.vu_blink_88@hcmut.edu.vn (P.D.V.); pldthuyet.sdh20@hcmut.edu.vn (T.L.D.P.); ptsnam@hcmut.edu.vn (N.T.S.P.); 2 Vietnam National University Ho Chi Minh City, Linh Trung Ward, Thu Duc District, Ho Chi Minh City 740010, Vietnam; 3 Ecole Nationale Supérieure de Chimie de Montpellier, ICGM, Univ. Montpellier, CNRS, ENSCM, 34090 Montpellier, France; phuoc@chalmers.se (P.H.H.); francesco.di-renzo@enscm.fr (F.D.R.)

**Keywords:** metal-organic framework, MOF-808, particle size, modulator, anionic trapping

## Abstract

This study presents a simple approach to prepare MOF-808, an ultra-stable Zr-MOF constructed from 6-connected zirconium clusters and 1,3,5-benzene tricarboxylic acid, with tailored particle sizes. Varying the amount of formic acid as a modulator in the range of 200–500 equivalents results in MOF-808 materials with a crystal size from 40 nm to approximately 1000 nm. Apart from the high specific surface area, a combination of a fraction of mesopore and plenty of acidic centers on the Zr-clusters induces a better interaction with the ionic pollutants such as K_2_Cr_2_O_7_ and anionic dyes. MOF-808 shows uptakes of up to 141.2, 642.0, and 731.0 mg/g for K_2_Cr_2_O_7_, sunset yellow, and quinoline yellow, respectively, in aqueous solutions at ambient conditions. The uptakes for the ionic dyes are significantly higher than those of other MOFs reported from the literature. Moreover, the adsorption capacity of MOF-808 remains stable after four cycles. Our results demonstrate that MOF-808 is a promising ideal platform for removing oxometallates and anionic dyes from water.

## 1. Introduction

Oxometallates and anionic dyes originating from industries such as steel production, textile manufacturing, and paint- and ink-making have emerged as one of the most significant sources of water pollution [1,2,3]. Their presence in large amounts in wastewater can cause serious health problems, such as allergies, cancer, and gene mutations [3,4]. To remove these hazardous anions from wastewater, technologies based on anion trapping have been introduced as effective methods due to their simplicity, efficiency, and ability to reduce toxic additives [1,5,6,7]. Significant efforts have been recently achieved in the preparation and application of organic and inorganic materials towards improving the anion trapping capacity; however, numerous challenges remain and need to be solved [7,8,9,10,11]. For example, unmodified activated carbon is well-known to be an efficient adsorbent of anionic dyes but it is nearly inactive for dichromate ions [12]. At the same time, some silica-based materials and inorganic composites can capture oxometallates well, but perform poorly in removing organic dyes [12]. Besides, high stability and facile recyclability of the materials in water are also important demands [6,13,14]. Introducing new materials as environmentally friendly and efficient adsorbents to remove oxometallates and anionic dyes from aqueous solutions is therefore highly essential [15]. 

**Figure 1:**
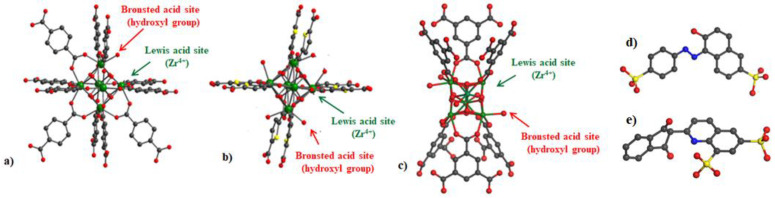
Representation of acidic sites, including Brønsted and Lewis acid sites, on Zr-cluster in UiO-66 framework (a typical 12-connected Zr-MOF) (**a**), DUT-67 framework (a typical 8-connected Zr-MOF) (**b**), and MOF-808 framework (a typical 6-connected Zr-MOF) (**c**), representative formulas of sunset yellow (**d**) and quinoline yellow (**e**). Color scheme: Zr (green); O (red); N (blue); S (yellow); C (grey).

Zirconium-based metal-organic frameworks have attracted much attention from scientists as promising materials for catalysis and adsorption during the past decade [16,17]. Apart from having the common characteristic of the MOF family, such as a large specific surface area and structural and chemical tunability, their outstanding performances derive from the defect positions, which are the open acidic sites (uncoordinated Zr-sites) [18,19]. Along with high hydrolytic stability, these positively charged sites in frameworks provide an ideal platform for capturing anions in water media [3,13,20]. In several recent reports, UiO-66 and its derivatives, which are typically 12-connected Zr-nets (MOFs with coordinatively saturated Zr-clusters), were widely employed to efficiently remove dichromate ions from aqueous media [21,22,23]. However, the accidental formation of defective sites is difficult to control and reproduce from batch to batch during the synthesis of these 12-connected Zr-MOFs (Figure 1a) [18]. Therefore, employing Zr-MOFs containing a low number of cluster connectivity (namely, 8- and 6-connected Zr-MOF) with inherent acidic centers in their frameworks could be a promising approach (Figure 1b,c) [2,3,24,25,26,27]. 

In this work, we demonstrate the efficiency of utilizing MOF-808, which is a representative 6-connected Zr-MOFs constructed from zirconium clusters and 1,3,5-benzenetricarboxylate linkers with an *spn* topology, for the removal of Cr(VI) anionic species, sunset yellow (Figure 1d), and quinoline yellow (Figure 1e) in aqueous solutions. To our knowledge, the study on anion adsorption by MOF-808 is rare in the literature. Besides, adjusting the size of the 6-connected Zr-MOF particles was investigated upon using formic acid as an efficient modulator for the crystallization of MOF-808, leading to the difference in its anion trapping capacity.

## 2. Materials and Methods

All reagents and starting materials were purchased from Sigma-Aldrich (Saint-Quentin-Fallavier, France) and were used as received without any further purification. 

### 2.1. Synthesis of MOF-808 Analogs 

In a typical procedure, a mixture of ZrOCl_2_·8H_2_O (0.81 g, 2.5 mmol) and 1,3,5-benzenetricarboxylic acid (0.18 g, 1.60 mmol) was dissolved in a mixture of DMF (47 mL) and HCOOH (28 mL, 0.75 mol, HCOOH/Zr^4+^ molar ratio = 300 equivalents). The resulting solution was sonicated for 5 min and then distributed into 4 vials (20 mL in volume) with tight caps. These vials were then placed in an oven at 80 °C for 120 h. The solid product was consequently collected by decanting the mother liquor and washing it with DMF (3 × 100 mL). The solid was further washed with acetone (5 × 100 mL) at 30 °C to exchange DMF. The resulting product was eventually activated at 120 °C for 6 h under a vacuum, giving 0.51 g of solid white powders of MOF-808 (Zr_6_O_5_(OH)_3_(BTC)_2_(HCOO)_5_(H_2_O)_2_, M = 1354 g/mol, 83% based on ZrOCl_2_·8H_2_O). This material was denoted as MOF-808_300.

The synthesis of other MOF-808 analogs was carried out using a similar protocol in which the amount of formic acid, as the modulator, was varied, including 200, 250, 350, 400, 450, and 500 equivalents (Table 1). The obtained materials were denoted as MOF-808_*x*, in which *x* is the number of equivalents.

### 2.2. Adsorption Studies

#### 2.2.1. Cr_2_O_7_^2−^ adsorption

The Cr_2_O_7_^2−^ adsorption experiments were carried out in an aqueous phase under ambient conditions. In a representative procedure, the activated MOF-808 (0.01 g) was added into 15 mL of the K_2_Cr_2_O_7_ solution with a concentration of 500 ppm (500 mg of K_2_Cr_2_O_7_/L, pH~4.6). The resulting mixture was then stirred for 120 min at 30 °C. After adsorption, the solid MOF material was separated by centrifugation (3000 rpm for 30 min). Because the pH- and concentration-dependent Cr_2_O_7_^2−^/CrO_4_^2−^ equilibrium could significantly affect the UV-Vis spectrophotometric result, the liquid phase was adjusted to a pH~4.6 by adding diluted acetic acid or ammonia solutions before performing the UV-Vis absorbance measurements. The Cr(VI) adsorption capacity was then quantified as the dichromate form based on the calibration curve of the potassium dichromate concentration vs. its absorbance at 361 nm at similar pH conditions, therefore independent from the equilibriums of the Cr(VI) forms. 

For the recycling test, the used MOF-808 was collected by centrifugation, washed intensively with water and acetone containing 5% of HCOOH, respectively, to remove adsorbates, and re-activated in a vacuum at 120 °C for 4 h, and then reused for a new adsorption experiment. 

#### 2.2.2. Dye Adsorption

To evaluate the dye adsorption capacity of MOF-808, two individual series of experiments using quinoline yellow (sodium 2-(1,3-dioxoindan-2-yl) quinolinedisulfonate) and sunset yellow (disodium 6-hydroxy-5-[(4-sulfophenyl) azo]-2-naphthalenesulfonate) as adsorbates were investigated. Similar to the study for K_2_Cr_2_O_7_, the fresh samples of MOF-808 were added to aqueous solutions containing 500 ppm of quinoline yellow and sunset yellow (15 mL, pH~6.8), respectively. The resulting mixtures were then stirred for 120 min under ambient conditions. The clear upper solutions were collected by centrifugation, and the pH value of the resulting aqueous phases was adjusted to ~6.8 by adding diluted acetic acid or ammonia solutions. The adsorbed amount of dye compounds was determined by the change in the corresponding solution concentrations using the UV-VIS absorbance measurements at 441 and 482 nm, respectively. 

To investigate the factors affecting the performance of the MOF-808 material, the adsorption conditions, including temperature, pH, time, and adsorbate concentration, were respectively varied.

### 2.3. Instruments

Nitrogen physisorption measurements were conducted using a Micromeritics 2020 volumetric adsorption analyzer system (Micromeritics, Norcross, GA, USA). Samples were pretreated by heating under a vacuum at 120 °C for 3 h. The equivalent specific surface area was calculated using the Brunauer-Emmett-Teller (BET) model in a relative pressure range of 0.01–0.10 p/p_0_ while the pore size distribution was assessed using the density functional theory (DFT) method based on slip modern.

X-ray powder diffraction (XRD) patterns were recorded using a Cu Kα radiation source on a D8 Advance Bruker powder diffractometer (Bruker AXS GmbH, Karlsruhe, Germany) with an Ni filter. The measurements were performed in a 2θ range of 2–30° with an angular step size of 0.01° and scanning rate of 0.6° per min. Particle size and cell parameter were average values calculated for five planes (111), (311), (222), (400), and (331) of XRD patterns. Peak position and full width at the half maximum were taken from a single peak fitting mode using FullProf software. Detailed information on the calculation can be found elsewhere [28].

Morphology of the MOF-808 samples was conducted by scanning electron microscopy (SEM) using a Hitachi S2600N microscope (Hitachi, Japan). Average size and size distribution were statistically determined by measuring the sizes of more than 100 particles using ImageJ software.

Thermal gravimetric measurements (TGA) were investigated using a TA Instruments SDT Q600 Thermal Gravimetric Analyzer (TA instruments, New Castle, DE, USA). In each experiment, the sample was placed in an alumina pan and heated from 40 to 900 °C with a ramping rate of 10 °C min^−1^ under 60 mL min^−1^ of airflow. 

Fourier transform infrared spectroscopy (FT-IR) measurements were recorded using a Bruker Alpha instrument (Bruker AXS GmbH, Karlsruhe, Germany) equipped with a diamond crystal. Each spectrum was accumulated from 32 scans at a resolution of 4 cm^−1^ recorded in the 4000–550 cm^−1^ range.

UV-Vis absorbance measurements were carried out with the Thermo Scientific G10S UV-Vis device (Thermo Scientific, Waltham, Massachusetts, USA). The concentrations of K_2_Cr_2_O_7_, sunset yellow, and quinoline yellow solution were determined based on the calibration curves of the concentrations of K_2_Cr_2_O_7_, sunset yellow, and quinoline yellow versus their absorbance recorded at 351, 481, 442 nm, respectively.

For the proton nuclear magnetic resonance (^1^H-NMR) analysis, the dried samples were digested using a small amount of CsF (15 mg) with 5 drops of deuterated hydrochloric acid (DCl, 37%) for 6 h. The ^1^H-NMR spectra of the resulting solution added with deuterated DMSO (DMSO-d_6_) were recorded by the Bruker spectrometer at 500 MHz (Bruker AXS GmbH, Karlsruhe, Germany). Chemical shifts (ppm) were referenced to tetramethylsilane (0.00 ppm).

## 3. Results and Discussion

### 3.1. MOF-808 Anologues Synthesis and Characterization

**Figure 2:**
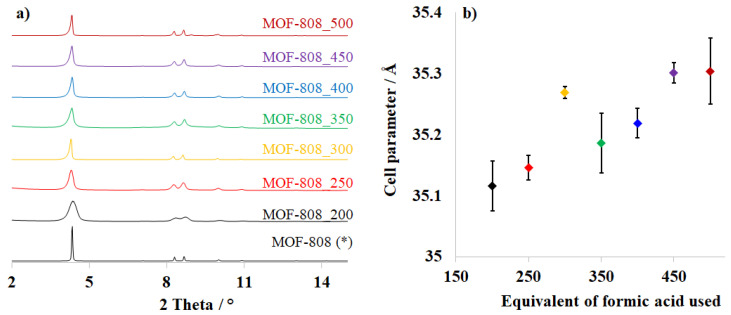
XRD patterns (**a**) and cell parameter (**b**) of as-made MOF-808 synthesized with different amounts of formic acid. * Theoretical pattern of MOF-808.

**Figure 3:**
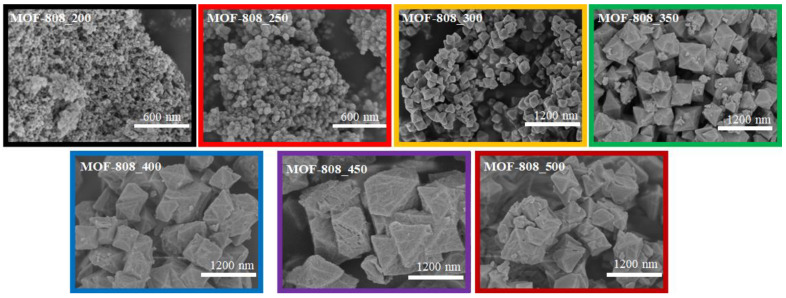
SEM images of MOF-808 synthesized with different amounts of formic acid.

The MOF-808 structure was constructed from 6-connected zirconium clusters and 1,3,5-benzenetricarboxylate molecules, affording a 3-D porous framework containing two different kinds of pores [25,26,27]. In earlier works, a huge amount of monocarboxylic acids or inorganic acids was employed to promote the formation of unsaturated Zr-clusters for further application in catalysis and adsorption. The presence of modulator molecules is of paramount importance in controlling the coordination geometry of Zr-cluster and subsequently significantly affecting the crystallinity, morphology, and crystal size of the zirconium-based MOFs [16,29]. In this work, the particle size of MOF-808 crystals was tuned by varying the amount of formic acid from 200 to 500 equivalents. The X-ray diffraction patterns of the resulting powders (Figure 2a) showed reflections at 2θ of 4.2. 8.2, 8.6, 10.0, and 10.9° with respect to planes (111), (311), (222), (400), and (331). These signature peaks were then employed to refine the unit cell parameters. The prepared materials crystallized in the cubic space group Fd$\bar{3}$m with the lattice parameters matched well with previously reported MOF-808 data [25,26,27]. Interestingly, an increase in modulator concentration resulted in a gradual enhancement of MOF-808 crystallinity and cell parameters (Figure 2b and Table 2), which expand from 35.12 to 35.31 Å. The shape and size of synthesized MOF-808 materials, which were observed on SEM images (Figure 3), were also improved as large amounts of formic acid were used in the synthetic phase.

The SEM image of MOF-808 synthesized with 300 equivalents of formic acid (MOF-808_300) exhibited regular octahedral crystals in the size of 300 nm (Figure 3 and Figure S3), while its BET specific surface area was up to 3291 m^2^/g (Entry 3_Table 2). However, the syntheses with less than 300 equivalents of HCOOH produced poor crystals (MOF-808_250 and MOF-808_200) with ill-defined morphology (Figure 3, Figures S1 and S2). The crystal size of these two samples was indeed significantly smaller than that of the MOF-808_300 (Figure 3 and Table 2), which was consistent with the above-described XRD results. The diameter of the crystals was approximately only 60 nm in the presence of 250 equivalents of HCOOH, while this value was below 40 nm when using 200 equivalents (Entry 1–2_Table 2 and Figure S1). The utilization of short-chain monocarboxylic acids as modulators has been usually preferred in the synthesis of Zr-MOFs [27]. These compounds are generally considered non-structural moieties, which temporarily bind to the metal precursor and are exchanged for carboxylate linkers without affecting the nets. By competing with organic building blocks, the formation of Zr-MOF nuclei, which are rapidly assembled from Zr^4+^ cations and carboxylate ligands, could be slowed down, controlling the growth of high-quality Zr-MOFs crystals with a larger size. In fact, by steadily increasing the formic acid amounts to 350 and 400 (MOF-808_350 and MOF-808_400), the crystal size could be grown up to approximately 600 and 700 nm (Figure 3, Figures S4 and S5). Furthermore, this value increased to approximately 1000 nm at 450 and 500 equivalents of HCOOH (Figure 3, Figures S6 and S7). Although an improvement in the crystal size was observed, the formation yield of MOF-808 decreased from 83% to 36% as the used formic acid amount increased from 300 to 500 equivalents (Table 1). Moreover, no desired product was found if 600 equivalents of this chemical were employed because of an over competition of the modulator compounds and organic building blocks.

**Figure 4:**
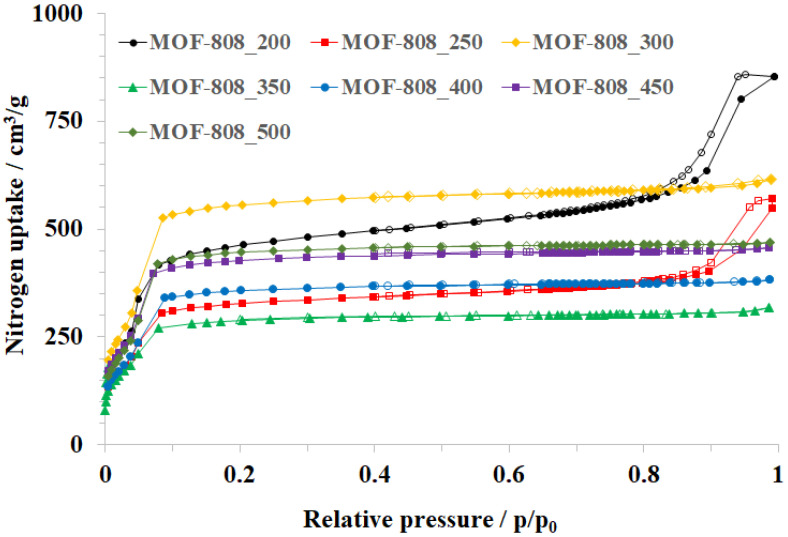
Nitrogen physisorption isotherms of MOF-808 samples synthesized with various amounts of formic acid.

**Figure 5:**
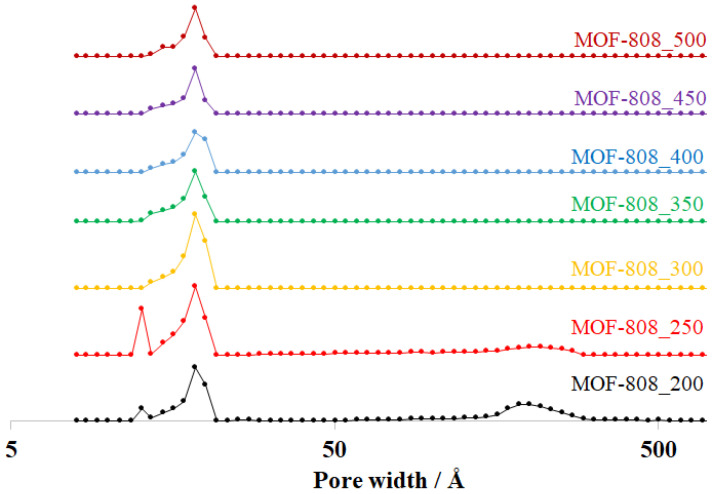
Pore size distribution of MOF-808 samples synthesized with various amounts of formic acid.

The modulation role of formic acid is of paramount importance in adjusting the size, shape, and crystallinity and, therefore, significantly impacts the textural properties of MOF-808. A notable change in the adsorption behavior of MOF-808 was observed when varying the formic acid amount in the synthesis phase. The MOF-808_250 sample shows a combination of type I and type IV isotherms (Figure 4), indicating the existence of both micro and mesoporous structures. Particularly, there are three different pore diameters in MOF-808_250, including 12.7 Å, 18.5 Å, and 216 Å (Figure 5). The mesoporous architectures in Zr-MOFs are attributed to the agglomeration of the nuclei of Zr-based MOFs, which were generated via the fast-rate reaction of Zr^4+^ cations with carboxylate ligands. In other words, the ratio of mesopores/micropores could be accelerated by using lower modulator amounts. The mesopore region was indeed improved as the amount of HCOOH was reduced to 200 equivalents, leading to a considerable improvement in nitrogen uptake from 570 to 873 cm^3^/g and surface area from 1627 to 2370 m^2^/g) (Figure 4 and Table 2).

By contrast, slowing down the reaction rate by increasing the HCOOH amount could facilitate the growth of highly crystalline and big crystals. The crystallinity of MOF-808 was improved significantly by employing 300 equivalents of HCOOH, leading to the appearance of ideal octahedral crystals with a diameter of about 300 nm instead of agglomerated crystals. The adsorption behavior of MOF-808_300 consequently performed a typical type I isotherm (Figure 4), which is assigned to a highly microporous system. Its pore diameter was determined to be about 18.7 Å (Figure 5), in good agreement with previous reports [25,26], and the surface area was achieved up to 3291 m^2^/g (Entry 3_Table 2). However, a steady increase in the formic acid amount from 300 to 350 equivalents caused a substantial drop in crystallinity and nitrogen uptake from 615 to 208 cm^3^/g (Figure 4). The generation of small MOF-808 nuclei, which was observed on the outer surface of octahedral crystals in the SEM image of the MOF-808_350 sample (Figure 2 and Figure 3), could be a rational reason for this reduction. It was suggested that increasing the concentration of the monocarboxylic acids as modulators in the synthesis phase could promote a reversal exchange, in which these molecules replaced carboxylate ligands on Zr-clusters [16,27]. However, such an exchange process could initiate the reconstruction of MOF-808 into new particles with higher crystallinity. This interesting phenomenon was indeed reported in the studies of Kaskel et al. on the HCl treatment for healing the crystallinity and porosity of DUT-67, a typical 8-connected Zr-MOF, [18,30]. Moreover, in this work, the self-repair of the material proceeded to achieve well-defined crystalline MOF-808 as 400 equivalents of formic acid were used for the synthesis of MOF-808. Its crystallinity could be nearly re-established when employing 500 equivalents of HCOOH as the modulator (Figure 2a). Together with the rejuvenation of crystallinity, the nitrogen adsorption ability of MOF-808_400, MOF-808_450, and MOF-808_500 were significantly improved to 380, 465, and 470 cm^3^/g, respectively. As a result, the BET surface area increased to 1853, 2279, and 2677 m^2^/g, respectively (Table 2). Notably, the MOF-808 unit cell was changed in the same trend as the used HCOOH amount was tuned in the range of 200–500 equivalents. In particular, the refined cell parameter was only about 35.15 Å when using 250 equivalents of HCOOH as a modulator, while at 300 equivalents this value could be approximately 35.27 Å. It then dropped to about 35.22 Å at 350 equivalents before retrieving 35.31 Å at 450 and 500 equivalents (Table 2). The structure and crystallinity of Zr-based metal-organic frameworks can self-adjust to adapt to the highly acidic environments, leading to the recovery of these features.

**Figure 6:**
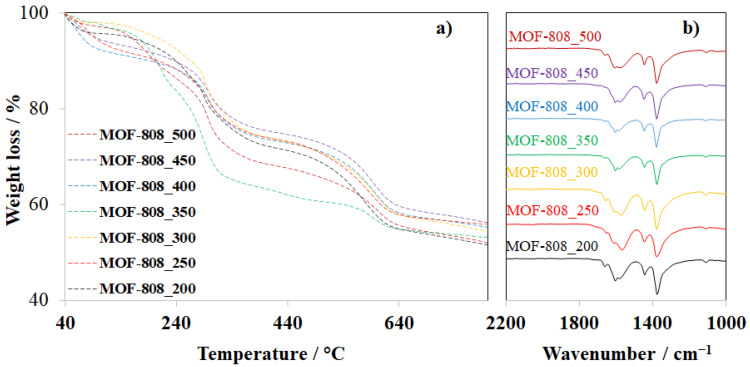
TGA results (**a**) and FT-IR spectra (**b**) of MOF-808 samples synthesized with various amounts of formic acid.

The thermogravimetric analysis (TGA) of activated MOF-808 samples was further investigated to take a look deep inside at the effect of crystal sizes on thermal stability. Interestingly, the TGA curves of all samples had similarities in the general shape with two major steps of decomposition at about 300 and 600 °C (Figure 6a). Although defective sites in Zr-MOF nets could lead to differences in TGA profiles because of missing linkers or clusters [22,31], the TGA results in this work confirmed that the phase purity of MOF-808 samples synthesized with various amounts of formic acid was quite similar. Therefore, it can be concluded that the crystal size of the MOF-808 materials had no significant effect on their thermal stability. In addition, the Fourier transform infrared (FT-IR) spectra of all MOF-808 samples, synthesized at the various concentrations of formic acid, exhibited no significant differences, which was confirmed by the similarity of the general shape in all of the FT-IR spectra of the MOF-808 samples (Figure 6b) [32].

### 3.2. Adsorption Studies

MOF-808 is well-known as a typical 6-connected zirconium metal-organic framework with strong acidic behavior [26]. The intrinsic existence of a large number of Lewis and Brønsted acid centers (namely, uncoordinated Zr-nodes and hydroxyl groups, respectively) in its structure offers strong affinities to negatively charged ions, such as oxometallates and dye anions (Figure 1c) [2,3,20,33]. In this work, the removal of Cr(VI) anionic species and anionic dyes, including sunset and quinoline yellow, from water using MOF-808 as an-anion trapping agent was investigated. First, a series of experiments were carried out in a broad range of pH values from 3.0 to 7.0 for K_2_Cr_2_O_7_ and 3.0 to 10.0 for organic dyes under ambient conditions. 

**Figure 7:**
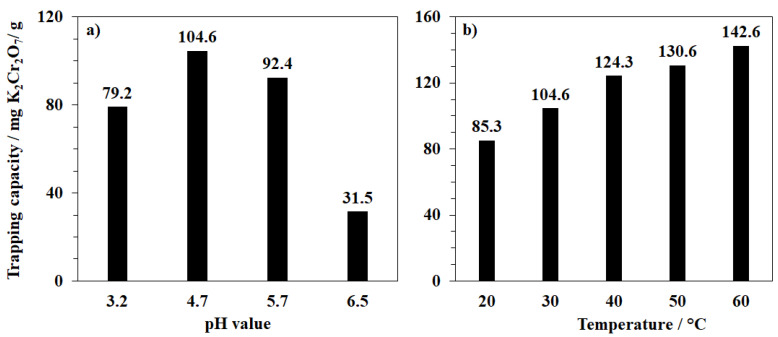
Effect of pH (**a**) and temperature (**b**) on K_2_Cr_2_O_7_ trapping capacity of MOF-808_300.

In the first study, the capturing anions in MOF-808 were strongly impacted by the presence of the competing protons and hydroxides in the aqueous solution, leading to significant changes in the trapping capacity at varied pH values. It should be noted that due to Cr_2_O_7_^2−^/CrO_4_^2−^ equilibrium, Cr(VI) anionic species in aqueous solutions could exist as Cr_2_O_7_^2−^, CrO_4_^2−^, or even HCrO_4_^-^ depending on both the pH value and Cr(VI) concentration [34]. However, the adsorption capacity, which is based on the variation of K_2_Cr_2_O_7_ amount in the solution before and after each experiment via the spectrophotometric measurements, would not be affected by this equilibrium because the pH of the liquid phase after adsorption was adjusted to approximate the value of the original solution [35]. In other words, although applying various pH conditions could lead to the change of Cr(VI) form for the absorption into MOF-808, all final adsorbed Cr(VI) amounts obtained in this study were quantified as the dichromate form. The highest value of 104.6 mg/g for trapping Cr_2_O_7_^2−^ ions was obtained at pH~4.7. Under less acidic conditions, namely pH~5.7 and 6.5, this value was reduced to 92.4 and 31.5 mg/g, respectively (Figure 7a). The removal efficiency of Zr-MOFs originates from the freedom of positively charged centers on the Zr clusters. In an aqueous phase, such Zr sites would be hydrated to form open Brønsted acid centers (Zr-O-H), which can attract anions [36]. High pH conditions can lead to deprotonation, decreasing the affinity of MOF-808 for anions. However, the adsorptive performance of MOF-808 for K_2_Cr_2_O_7_ decreased to 79.2 mg/g at pH~3.2 (Figure 7a). Further acidifying the solution hindered the process as the plenty of the protons in water could directly compete with the acidic centers on Zr-clusters in trapping Cr_2_O_7_^2−^ ions. Consequently, the anions remained in the liquid phase instead of being attracted to Zr-nodes. Importantly, the form of Cr(VI) anionic species in aqueous solutions (Cr_2_O_7_^2−^, CrO_4_^2−^, or HCrO_4_^−^) also impacted the performance of MOF-808 due to the differences in their mass, structure, and electric charge. Therefore, applying various pH conditions to the adsorption could lead to changes in not only the active MOF-808 sites but also the Cr(VI) form, obviously affecting the trapping capacity.

**Figure 8:**
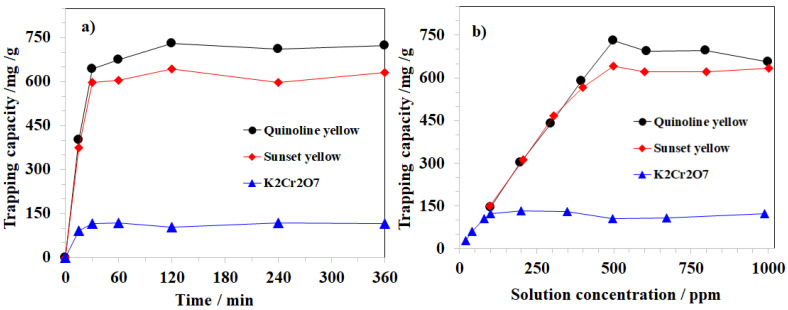
Effect of adsorption time (**a**) and solution concentration (**b**) on K_2_Cr_2_O_7_, quinoline yellow, and sunset yellow trapping capacity of MOF-808_300.

The effect of the adsorption time on MOF-808_300 performance was also investigated at 30 °C in various time intervals from 15 to 360 min. Consequently, the K_2_Cr_2_O_7_ trapping capacity of MOF-808_300 was recorded to be about 92.1 mg/g after 15 min. This value improved to about 104.6 mg/g at 120 min and remained almost the same over the further 240 min (Figure 8a). In addition to this, the experiment employing the different K_2_Cr_2_O_7_ concentrations was carried out to confirm the maximum adsorption capacity of MOF-808_300 under our experimental conditions. The equilibrium state was achieved to approximately 105 mg of K_2_Cr_2_O_7_/g as the concentration of the initial solution was approximately 80 ppm. Further increasing the initial K_2_Cr_2_O_7_ concentration to 500 ppm did not lead to any improvements in the adsorption capacity of MOF-808_300 (Figure 8b). 

The study on the effect of temperature on the adsorption process showed that the K_2_Cr_2_O_7_ trapping capacity of MOF-808 could be significantly improved by increasing the temperature. At 60 °C, an enhancement of ~50% in the adsorption of K_2_Cr_2_O_7_ could be achieved as compared to the experiment at 30 °C, suggesting that this process was endothermic (Figure 7b), in good agreement with previous studies on TiO_2_-MCM-41 [37], VO_2_ nanoparticles [38], activated biochars [39,40], ZIF-67 and BUC-17 (cobalt-based metal-organic frameworks) [41,42], and UiO-66-NH_2_ (zirconium-based metal-organic frameworks) [43]. This enhancement in adsorption capacity could be attributed to the generation of new adsorption sites or the increase in intraparticle diffusion rate of Cr(VI) ions into the material pores at an elevated temperature [37]. Indeed, in our study, an extra region of mesopore from 180–500 Å was observed for the pore size distribution of the reused MOF-808 sample, apart from the main pore distribution of approximately 18.7 Å as observed for the fresh sample. This pore extension could be due to the reaction between the Cr(VI) anion and the organic parts in the MOF material. It is obvious that the interaction between highly oxidative Cr(VI) species and the absorbent should be assessed, and the Cr(VI) assumption related to such redox reactions could not be excluded from the adsorption study.

Utilizing Zr-MOFs with reduced cluster connectivity is a brilliant approach for the removal of anions from water. In previous studies, the idealized 12-connected Zr-MOFs, namely UiO-66, showed a modest performance with only 8.8 mg of K_2_Cr_2_O_7_/g (Entry 1_Table 3). Its trapping capacity could slightly increase to 34.42 and 75.5 mg/g, as amino and hydroxyl groups were respectively present on benzene dicarboxylate linkers (Entry 2–3_Table 3) [23]. Besides, loading UiO-66 on carriers containing multi-functional groups, such as alginic acid (UiO-66-HA) and modified cellulose fibers (UiO-66-NH_2_@SiO_2_), could also improve the removal of oxometallates to approximately 130 mg K_2_Cr_2_O_7_ per gram of these composite materials (Entry 5–6_Table 3) [21,44]. Another potential strategy to improve Cr(VI) adsorption is employing defective Zr-MOFs. Very recently, Luis et al. found that the trapping capacity of UiO-66 could be increased by approximately three times from 8.8 to 22.4 mg of K_2_Cr_2_O_7_/g if the defective sites in the UiO-66 structure enhance from 15% to 25% (Entry 1–2_Table 3) [23]. Liu et al. have also introduced a new tetradentate pyrazine linker, which could bond with Zr-clusters for the formation of a novel 4-connected Zr-MOF (JLU—MOF60). This material performed an excellent trapping capacity of 149 mg/g (Entry 7_Table 3) [45]. However, using such new linkers or modifying ligands with multi-functional groups, or controlling the formation of defective sites generally remains difficult in the synthesis of Zr-based MOFs. Meanwhile, MOF-808, simply assembled from the commercially available 1,3,5-benzene tricarboxylic acid and zirconium salts, could trap up to 104.6 mg /g at 30 °C. This advantage opens up a great opportunity to employ MOF-808 as an efficient trapper for the removal of K_2_Cr_2_O_7_ in practical application. 

**Figure 9:**
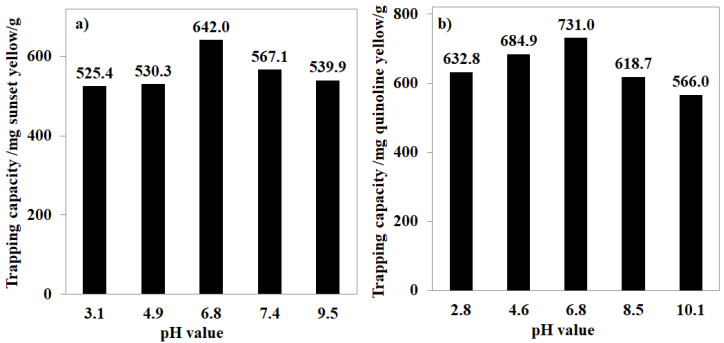
Effect of pH on sunset yellow (**a**) and quinoline yellow (**b**) trapping capacity of MOF-808_300 at 30 °C.

**Figure 10:**
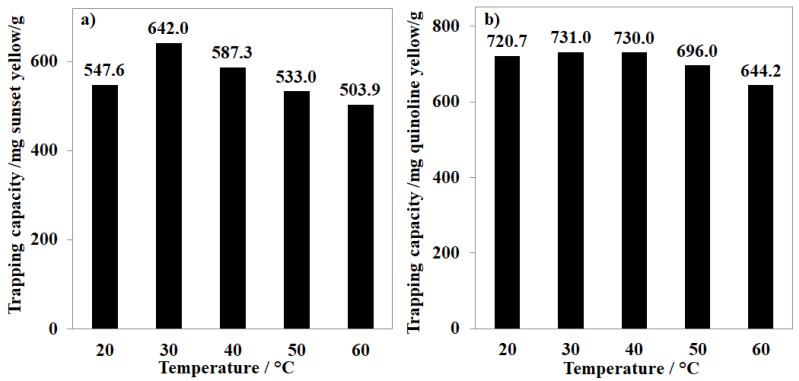
Effect of temperature on sunset yellow (**a**) and quinoline yellow (**b**) trapping capacity of MOF-808_300 at 30 °C.

To extend the application scope of MOF-808, the removal of anionic dyes, including sunset yellow and quinoline yellow, was also investigated in the broad range of pH and temperature. Varying the pH value of the aqueous solution led to significant changes in the performance of MOF-808 in trapping the anionic dyes. Particularly, each gram of MOF-808 could remove up to 642.0 mg of sunset yellow and 731.0 mg of quinoline yellow at pH~6.8 and 30 °C (Figure 9a,b). The adjustment to more acidic or basic conditions was found to be unnecessary, with gradual losses in the efficiency recorded for both cases of sunset and quinoline yellow. These results demonstrated that trapping anions of MOF-808 is sensitive to the pH of the solution and therefore needs to be intensively studied for each absorbate. In contrast to the case of K_2_Cr_2_O_7,_ in which the trapping capacity increased with the temperature, the adsorption of MOF-808 for sunset yellow and quinoline yellow gradually decreased to 503.9 and 644.2 mg/g, respectively, at 60 °C (Figure 10a,b). Notably, the effect of adsorption time and initial concentration of organic dye solutions on the adsorption of MOF-808_300 was also investigated at 30 °C. Our experimental results showed that the equilibrium state of MOF-808_300 could be obtained after 120 min for both quinoline yellow and sunset yellow at the initial concentration of 500 ppm with trapping capacities of 731.0 and 642 mg/g, respectively. No significant capacity enhancements were observed upon expanding the processing time and the dye concentration (Figure 8). 

Some popular MOFs with open metal sites, including MOF-199 (open copper centers) and MIL-101 (open iron centers), have been recently employed to remove sunset and quinoline yellows from water; however, these materials exhibited adsorption capacities of: 65.4 mg of quinoline yellow/g of MOF-199 and 81.3 mg of sunset yellow/g of MIL-101 at ambient temperature (Entry 9–10_Table 3) [46,47,48]. These uptake values are 7-fold and 12-fold lower than those of sunset yellow and quinoline, respectively, by the MOF-808_300 in the present work. Poor performances of MOF-199 and MIL-101 perhaps derive from the oversensitivity of the open metal centers, which are easier to be blocked by water molecules than to capture dye anions [49]. For comparison purposes, a commercial activated carbon was used as an adsorbent for the adsorption of quinoline yellow and sunset yellow under the same conditions as performed on MOF-808_300. The activated carbon showed an adsorption capacity of about 97 mg/g for both organic dyes (Entry 12_Table 3), which were still significantly lower than those of the MOF-808_300. Obviously, a MOF-808 with a 6-connected Zr-cluster offers a better platform with a superior adsorption capacity for anion trapping in an aqueous solution. 

**Figure 11:**
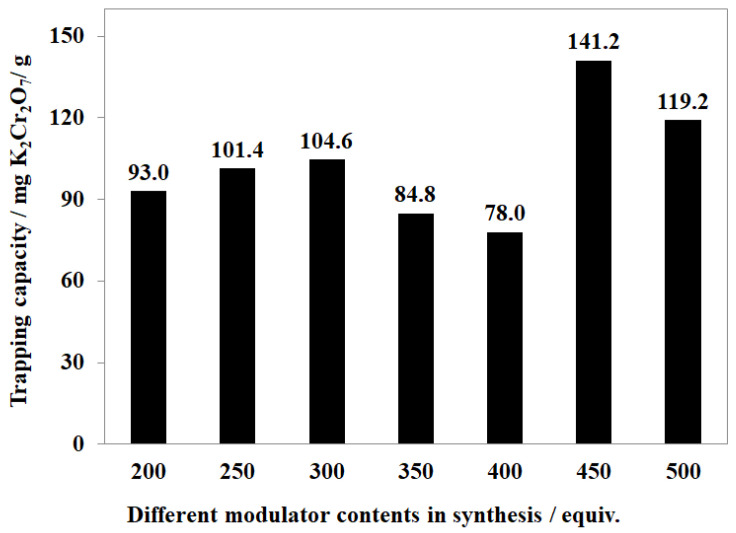
K_2_Cr_2_O_7_ trapping capacity of different MOF-808 samples synthesized with various formic acid concentrations in the synthesis phase.

The nature of MOF-808 crystals also played a vital role in Cr (VI) trapping from an aqueous solution. In this work, the morphology of MOF-808, particularly shape and dimension, was tuned by altering the formic acid amount in the synthesis phase from 200 to 500 equivalents with 50 equivalents for an increasing step. This rising of modulator content resulted in an improvement in MOF-808 crystal size from about 40 nm to approximately 1000 nm. Moreover, the anion trapping capacity of MOF-808 was generally affected by elevating the quantity of modulator used in the synthesis. The adsorption capacity for K_2_Cr_2_O_7_ was approximately 93 mg/g for MOF-808-200, while it increased to 101.4 and 104.6 mg/g for MOF-808_250 and MOF-808_300, respectively (Figure 11). Furthermore, the maximum K_2_Cr_2_O_7_ trapping adsorption of 141.2 mg/g for was achieved with MOF-808_450. However, by using up to 500 equivalents of formic acid to synthesize MOF-808, a slight decrease in K_2_Cr_2_O_7_ trapping capacity was seen, although a modest increase in BET surface area was found from 2279 to 2677 m^2^/g. On the other hand, the removal efficiencies of MOF-808_350 and _400 were only 84.8 and 78.0 mg of K_2_Cr_2_O_7_/g (Figure 11), respectively. The crystal sizes of MOF-808_350 and MOF-808_400 were approximately 600 nm and 700 nm, respectively, but the significant loss of crystallinity and the surface area could be a rational reason for the unexpected reduction in the K_2_Cr_2_O_7_ removal.

**Figure 12:**
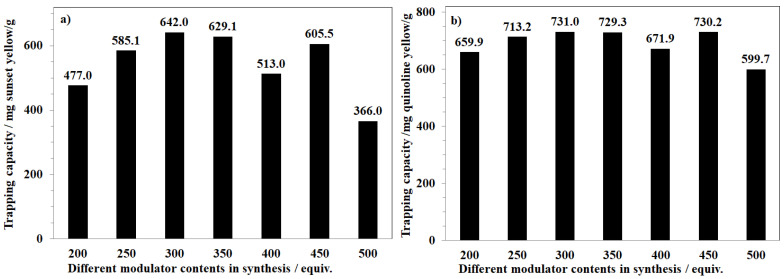
Sunset yellow (**a**) and quinoline yellow (**b**) trapping capacity of different MOF-808 samples synthesized with various formic acid concentrations in the synthesis phase.

Similarly, the anionic dyes trapping was significantly affected by the size of MOF-808, which was synthesized with different amounts of formic acid used in the synthesis phase. This dependency was found to have less of an impact on quinoline yellow than sunset yellow. The removal efficiency was generally improved if MOF-808 was prepared with a large amount of formic acid; it achieved up to 642.0 mg for sunset yellow and 731.0 mg for quinoline yellow when using one gram of MOF-808_300 as an absorbent (Figure 12a,b). However, the samples with more than 350 equivalents of formic acid as a modulator showed unexpectedly poor performances. The lowest trapping capacities were recorded for the case of MOF-808_500 with only 366.0 mg of sunset yellow/g and 599.7 mg of quinoline yellow/g, respectively (Figure 12a,b). This gradual decrease could be related to the absence of the mesopore region, which was not observed on the pore size distribution results of MOF-808 synthesized with more than 350 equivalents of formic acid. To clarify this interesting phenomenon, further investigations regarding the formation of defective sites in frameworks should be carried out. 

**Figure 13:**
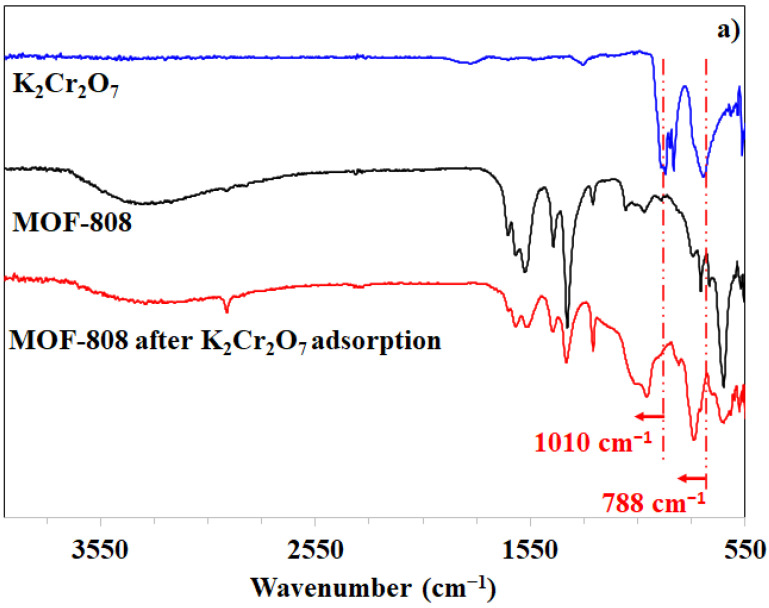

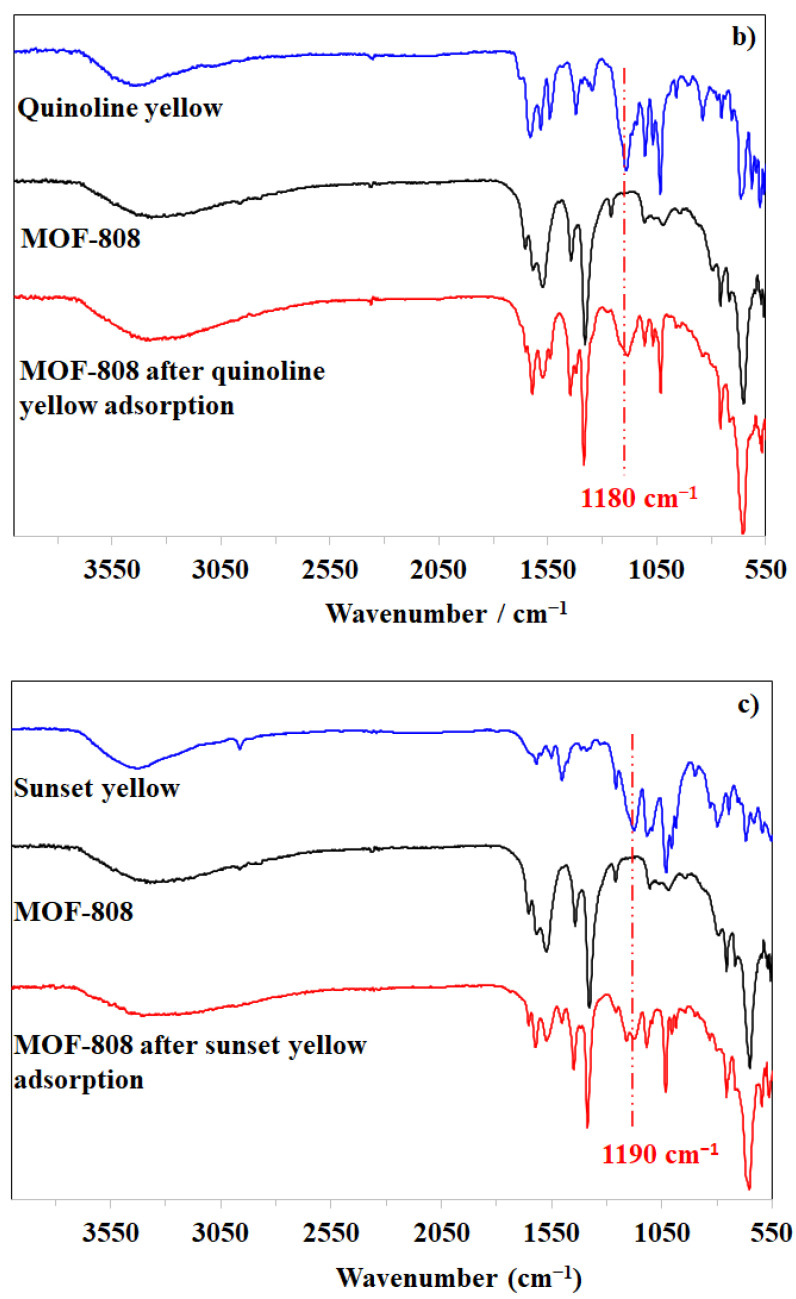
FT-IR spectra of MOF-808 samples after capturing K_2_Cr_2_O_7_ (**a**), quinoline yellow (**b**), and sunset yellow (**c**).

Regarding the presence of adsorbates in MOF-808, various techniques, including FT-IR, ^1^H-NMR spectroscopy, and PXRD, were employed to characterize the material after the aqueous adsorption. Particularly, the solid sample was collected by centrifugation and had its residual water removed under a reduced pressure at 60 °C for 6 h. As can be expected, the FT-IR spectrum of MOF-808 used for trapping K_2_Cr_2_O_7_ showed additional vibrations at 1010 and 788 cm^−1^ assigned to the asymmetric stretch of the (CrO_3_) group which was respectively observed at 925 and 735 cm^−1^ on the FT-IR spectrum of K_2_Cr_2_O_7_ (Figure 13a). This shift could be rationalized by the interaction of Cr_2_O_7_^2−^ anions and Zr^4+^ sites in the MOF-808 framework [32]. For organic dyes, their corresponding appearances were also found on the spectrum of the used MOF-808 via the presence of signature peaks at 1185 cm^−1^, which was attributed to the C-O stretch (Figure 13b,c). The corresponding ^1^H signals of the sunset and quinoline yellow dyes were observed on the ^1^H NMR spectra of the digested MOF-808 samples, respectively (Figures S8 and S9).

**Figure 14:**
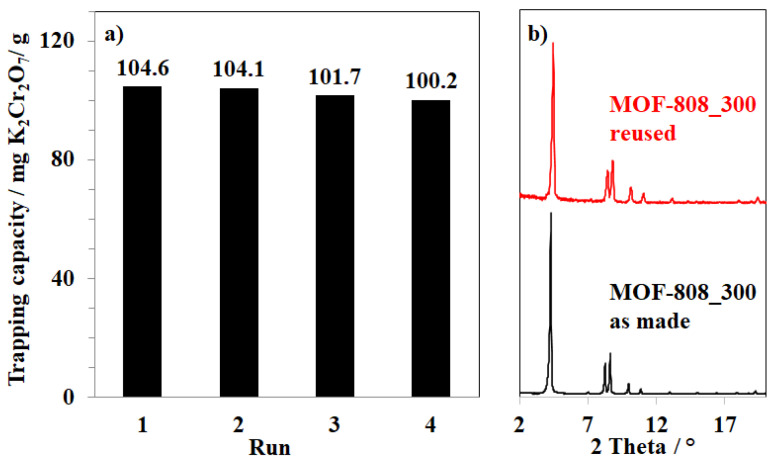
(**a**) Recycling test and (**b**) PXRD results of as made MOF-808_300 and MOF-808_300 after the fourth runs.

The ability to recover and reuse MOF-808 material for anion trapping is one of the key factors for practical applications. After the first cycle, the MOF-808_300 was collected by centrifugation and subsequently washed with DMF and acetone containing 5% of HCOOH to remove adsorbates and dried in a vacuum at 120 °C for 4 h. The regenerated material was consequently used for a new cycle of the adsorption of K_2_Cr_2_O_7_ at 30 °C. The adsorption capacity of the MOF-808_300 for K_2_Cr_2_O_7_ only decreased less than 5%, from 104.6 to 100.2 mg/g (Figure 14a). Considering that a small fraction of material could be lost during the regeneration step, it would be possible to imply that the MOF-808_300 could be recovered and reused at least three times without any significant degradation. 

**Figure 15:**
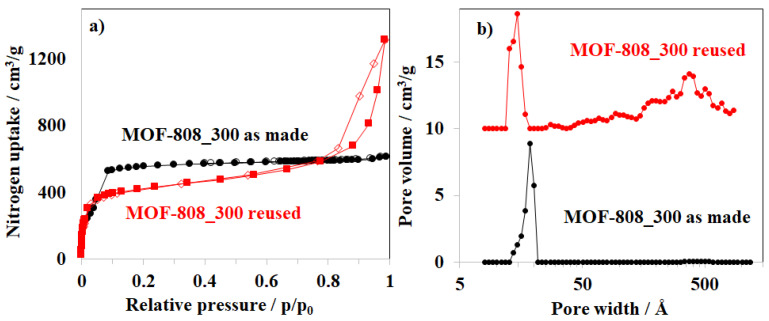
(**a**) Nitrogen physisorption isotherms and (**b**) pore size distribution of as made MOF-808 and MOF-808 after the fourth use.

The PXRD patterns of the materials after the first and fourth uses are consistent with that of the fresh MOF-808 sample, indicating that the crystallinity of MOF-808 has remained after the fourth cycle (Figure 14b, Figures S10 and S11). However, the nitrogen adsorption behavior of the reused sample slightly changed from the typical type I isotherm to the type IV isotherm with hysteresis loops, indicating the appearance of mesoporous structures (Figure 15a). Particularly, a region of mesopore from 180–500 Å was observed on the pore size distribution of the reused sample (Figure 14b), and a reduction in surface area was recorded from 3291 to 1481 m^2^/g (Entry 8_Table 2). These results showed that the pore walls in the MOF-808 framework were slightly broken up during the processes of adsorption and regeneration. 

## 4. Conclusions

The stable zirconium metal-organic frameworks with reduced Zr-cluster connectivity offers many great opportunities for practical applications. In our work, the 6-connected Zr-MOF, MOF-808, was demonstrated to be an efficient platform for removing Cr(VI) anionic species and anionic dyes, including sunset and quinoline yellows, in an aqueous solution. The high adsorption efficiency for those ionic species on MOF-808 in an aqueous solution depends on not only the properties of MOF-808, such as a high specific surface area, large pore size, and open Brønsted acid centers but also the adsorption conditions, e.g., pH, temperature, time and adsorbate concentration. The superior adsorption capacity of MOF-808 was recorded up to 141.2, 642, and 731 mg/g for K_2_Cr_2_O_7_, sunset yellow, and quinoline yellow, respectively, in a mild acidic aqueous solution at 30 °C. This anion trapping ability of MOF-808 was found to be significantly reliant on its morphology, including particle size and crystallinity. A simple and benign approach to tune the size of the MOF-808 crystal from about 40 nm to approximately 1000 nm was introduced by varying the amount of modulator (formic acid) in the synthesis route. In addition, the high uptake of the MOF-808 for Cr(VI) anions adsorption remained after four cycles, implying that this material is a promising recyclable adsorbent for the adsorptive removal of Cr(VI) anionic species and anionic dyes. 

## Figures and Tables

**Table 1:** Synthesis conditions of MOF-808 with different formic acid amounts.

No.	Sample Name	Formic Acid Amount (Equivalents)	Volume of Formic Acid (mL)	Volume of DMF (mL)	Mass of Product (g)	Yield of Reaction (%)
1	MOF-808_200	200	19	56	0.34	55
2	MOF-808_250	250	24	51	0.47	76
3	MOF-808_300	300	28	47	0.51	83
4	MOF-808_350	350	33	42	0.45	73
5	MOF-808_400	400	38	37	0.42	68
6	MOF-808_450	450	43	32	0.36	59
7	MOF-808_500	500	47	28	0.22	36

**Table 2:** Characteristic properties of MOF-808 synthesized with various amounts of formic acid.

No.	Sample Name	Formic Acid Amount (Equivalent)	Cell Parameter ^1^ (Å)	Crystal Diameter ^2^ (nm)	BET Surface Area (m^2^/g)	Average Pore Diameter ^3^ (Å)	V Total Pore ^3^ (cm^3^/g)	V Micropore ^3^ (cm^3^/g)
1	MOF-808_200	200	35.12 ± 0.04	<40	2370	23.9	1.18	0.59
2	MOF-808_250	250	35.15 ± 0.02	~60	1627	21.9	0.80	0.44
3	MOF-808_300	300	35.27 ± 0.01	~300	3291	18.7	0.82	0.79
4	MOF-808_350	350	35.19 ± 0.05	~600	1464	19.1	0.41	0.4
5	MOF-808_400	400	35.22 ± 0.02	~700	1853	18.7	0.51	0.50
6	MOF-808_450	450	35.30 ± 0.02	~1000	2279	18.6	0.60	0.60
7	MOF-808_500	500	35.31 ± 0.05	~1000	2677	17.8	0.63	0.62

^1^ Cell parameter was determined based on XRD patterns of MOF-808 analogs. ^2^ The diameters of MOF-808 samples were observed on SEM images. ^3^ The pore size diameters and pore volumes were calculated by the DFT method.

**Table 3:** Comparison of the anion trapping capacity of MOF-808 with various materials reported from the literature.

No.	Sample Name	K_2_Cr_2_O_7_ Capacity (mg/g)	Sunset Yellow Capacity (mg/g)	Quinoline Yellow Capacity (mg/g)	Ref.
1	UiO-66 with 15% missing-linker defects	8.8	-	-	[23]
2	UiO-66 with 25% missing-linker defects	22.4			[23]
3	UiO-66-NH_2_	34.4	-	-	[23]
4	UiO-66-(OH)_2_	75.5	-		[23]
5	UiO-66-HA	129.0	-		[44]
6	UiO-66-NH_2_@SiO_2_	137.0	-	-	[21]
7	JLU—MOF60	149.0	-	-	[45]
8	Mesoporous silica nanoparticles	42.2	-	-	[7]
9	MOF-199	-	-	65.4	[46]
10	MIL-101@graphene oxide	-	81.3	-	[47]
11	MOF-5@activated carbon	-	-	21.2	[48]
12	Activated carbon	No adsorption	96.0	97.0	This work
13	MOF-808	78.0–141.2	366.0–642.0	659.9–731.0	This work

## Data Availability

Data is contained within the article and Supplementary Materials.

## Supplementary Materials

The following are available online at https://www.mdpi.com/article/10.3390/nano11061398/s1, Figures S1–S7: SEM images of MOF-808 synthesized with different equivalents of formic acid, Figures S8–S9: digested NMR of MOF-808 and its analogues, Figure S10: XRD of reused MOF-808, Figure S11: photographs of the samples before and after the adsorption processes.
