# Supplementary material for: Efficient Removal of Chromium(VI) Anionic Species and Dye Anions from Water Using MOF-808 Materials Synthesized with the Assistance of Formic Acid
Source: Nanomaterials (Basel). 2021 May 25;11(6):1398. doi: 10.3390/nano11061398 (PMC8226478; doi:10.3390/nano11061398)
Supplement: Supplementary file 1 [file nanomaterials-11-01398-s001.zip › nanomaterials-1191924-supplementary.pdf]

Supplementary Materials

Efficient Removal of Chromium(VI) Anionic Species and Dye Anions from Water Using MOF-808 Materials Synthesized with the Assistance of Formic Acid

Khoa D. Nguyen [1,2,*], Phuoc H. Ho [3,†], Phuong D. Vu [1,2], Thuyet L. D. Pham [1,2], Philippe Trens [3,*], Francesco Di Renzo [3], Nam T. S. Phan [1,2] and Ha V. Le [1,2,*]

1 Department of Chemical Engineering, Ho Chi Minh University of Technology, 268 Ly Thuong Kiet Street, District 10, 740010 Ho Chi Minh City, Vietnam; phuong.vu_blink_88@hcmut.edu.vn (P.D.V.); pldthuyet.sdh20@hcmut.edu.vn (T.L.D.P.); ptsnam@hcmut.edu.vn (N.T.S.P.)
2 Vietnam National University Ho Chi Minh City, Linh Trung Ward, Thu Duc District, 740010 Ho Chi Minh City, Vietnam
3 Ecole Nationale Supérieure de Chimie de Montpellier, ICGM, Univ. Montpellier, CNRS, ENSCM, 34090 Montpellier, France; phuoc@chalmers.se (P.H.H.); francesco.di-renzo@enscm.fr (F.D.R.)
* Correspondence: khoand1989@hcmut.edu.vn (K.D.N.); philippe.trens@enscm.fr (P.T.); lvha@hcmut.edu.vn (H.V.L.)
† Present address: Chemical Engineering, Competence Centre for Catalysis, Chalmers University of Technology, SE-412 96 Gothenburg, Sweden.

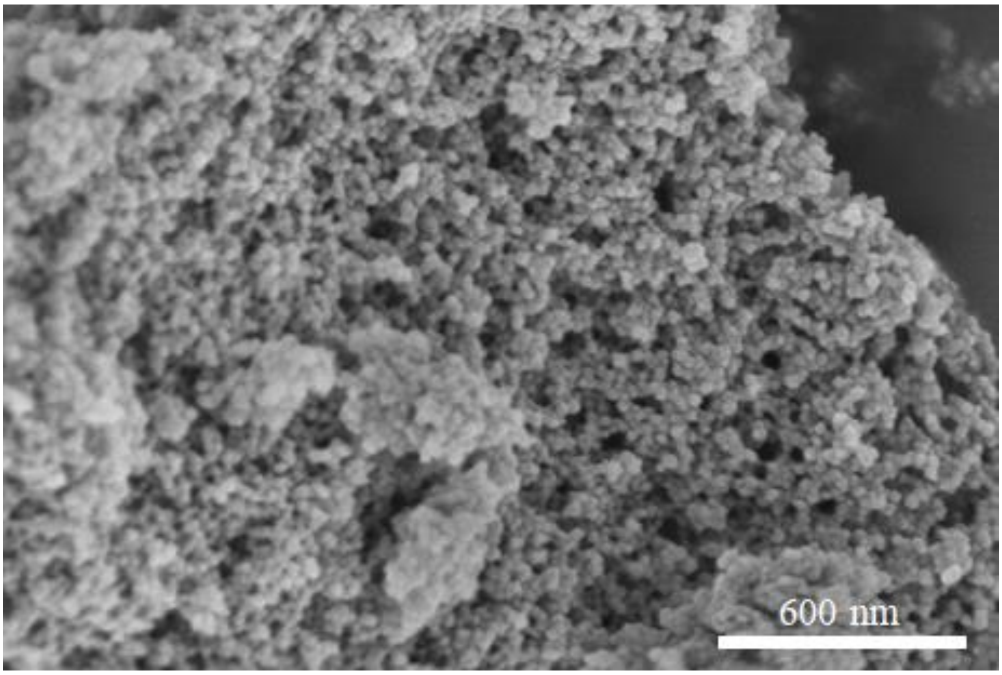

Figure S1. SEM image of MOF-808 synthesized with 200 equivalents of formic acid.

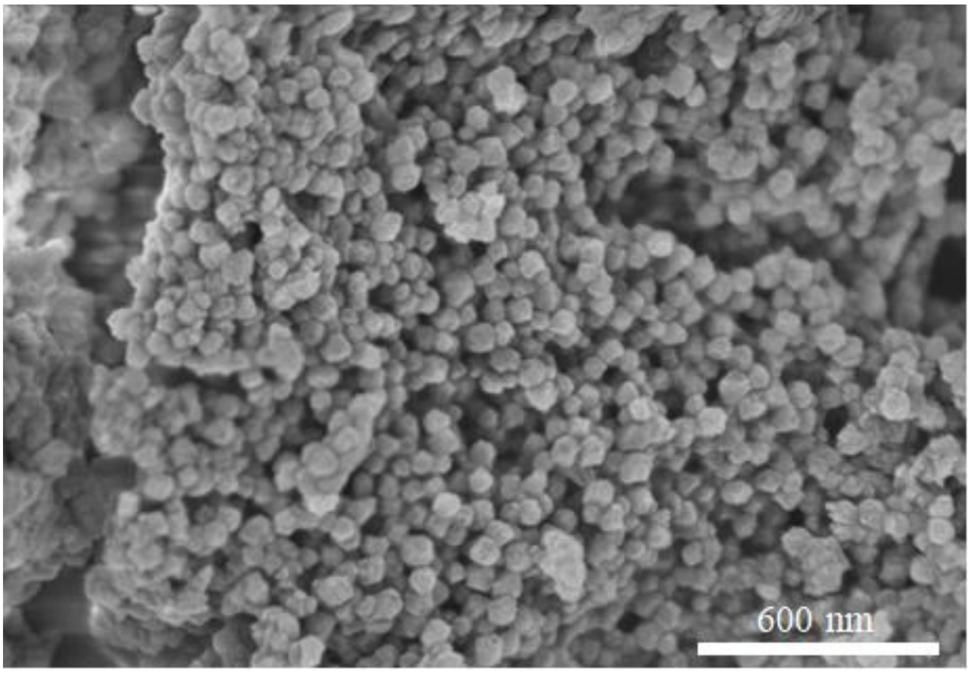

Figure S2. SEM image of MOF-808 synthesized with 250 equivalents of formic acid.

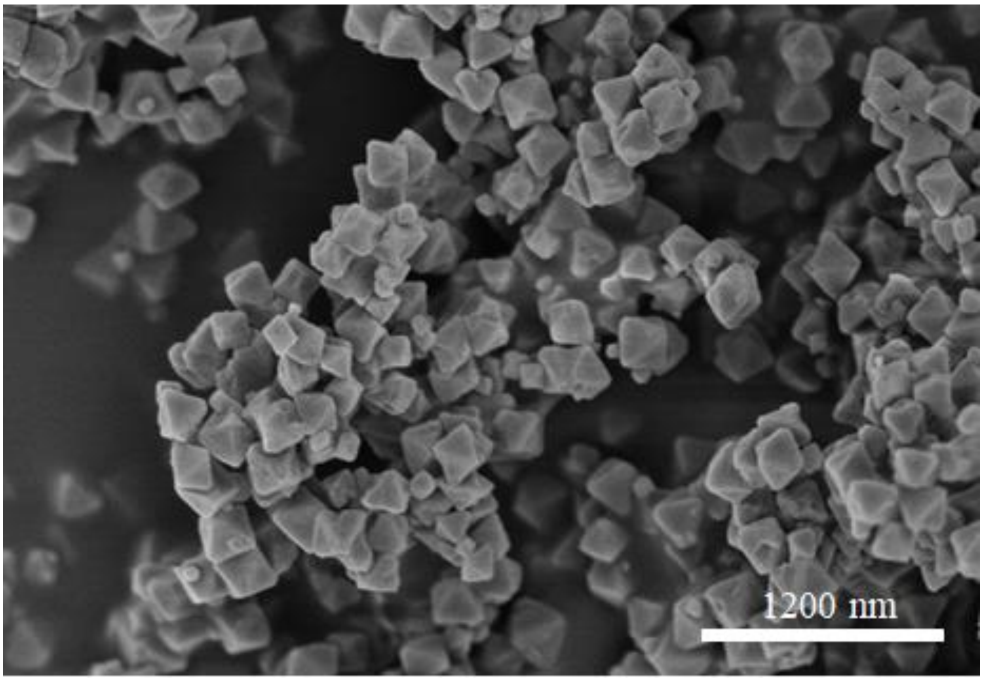

Figure S3. SEM image of MOF-808 synthesized with 300 equivalents of formic acid.

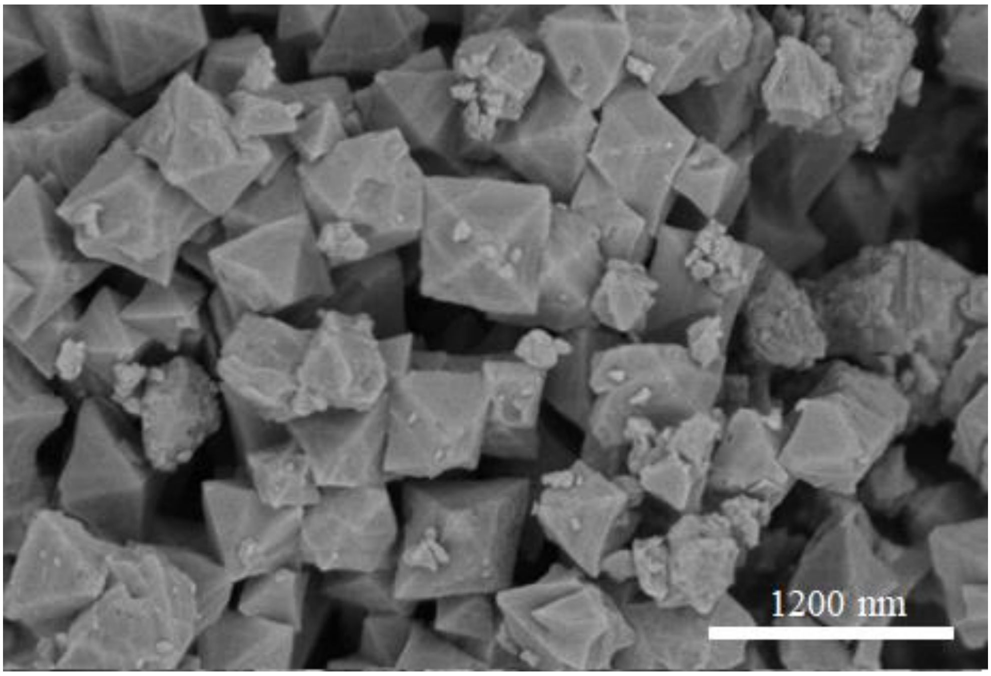

Figure S4. SEM image of MOF-808 synthesized with 350 equivalents of formic acid.

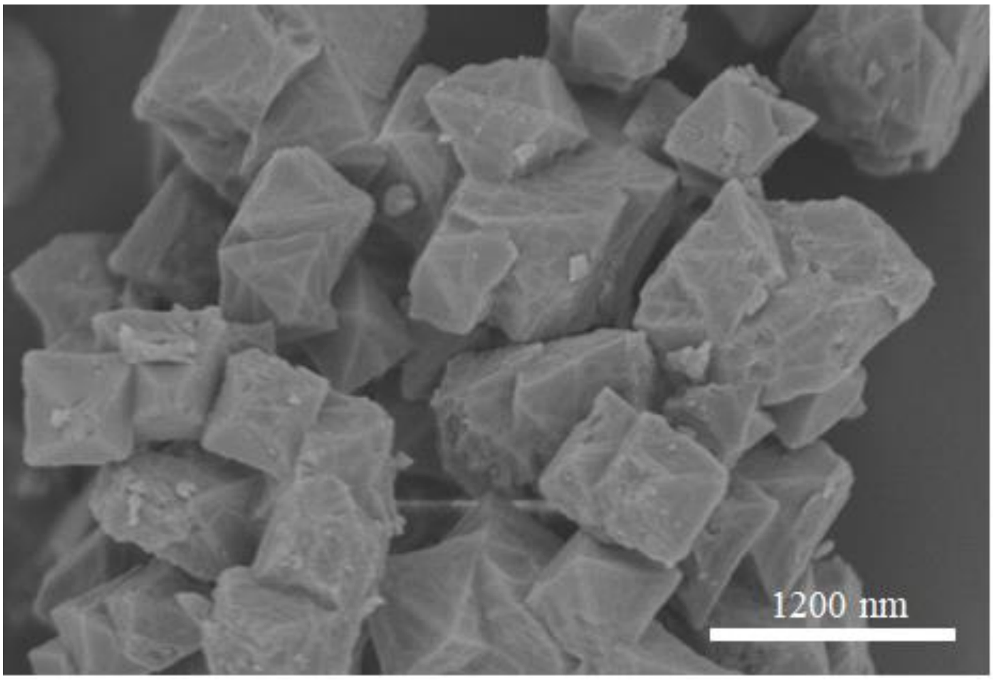

Figure S5. SEM image of MOF-808 synthesized with 400 equivalents of formic acid.

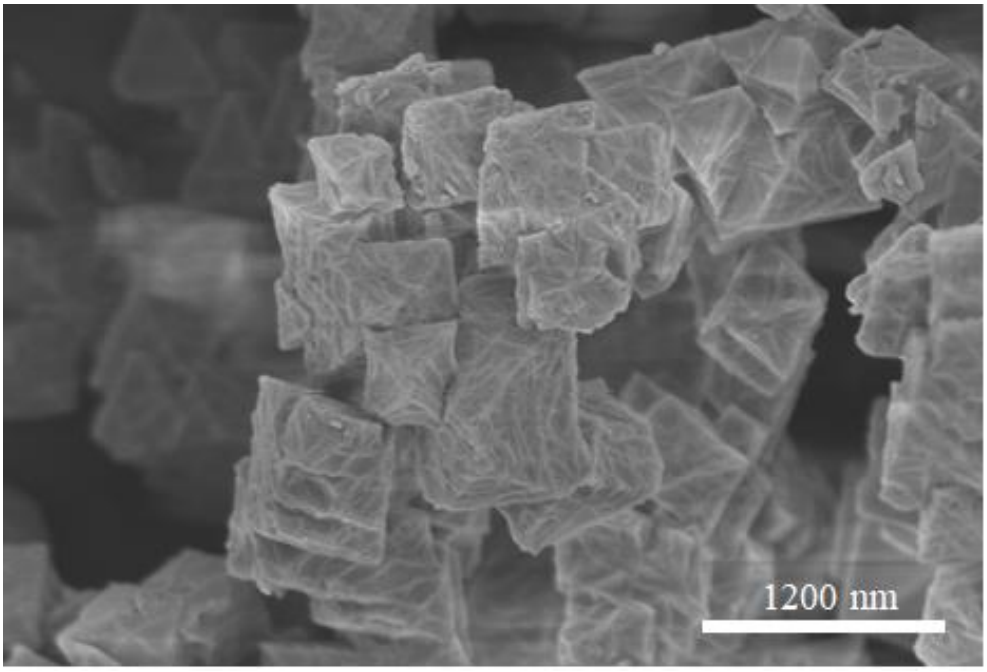

Figure S6. SEM image of MOF-808 synthesized with 450 equivalents of formic acid.

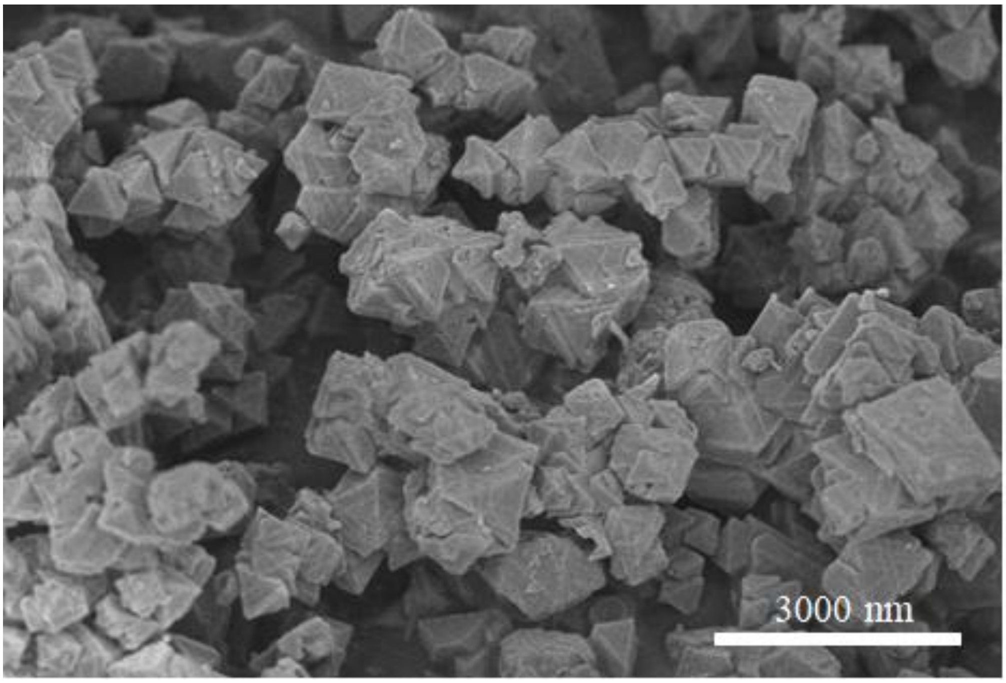

Figure S7. SEM image of MOF-808 synthesized with 500 equivalents of formic acid.

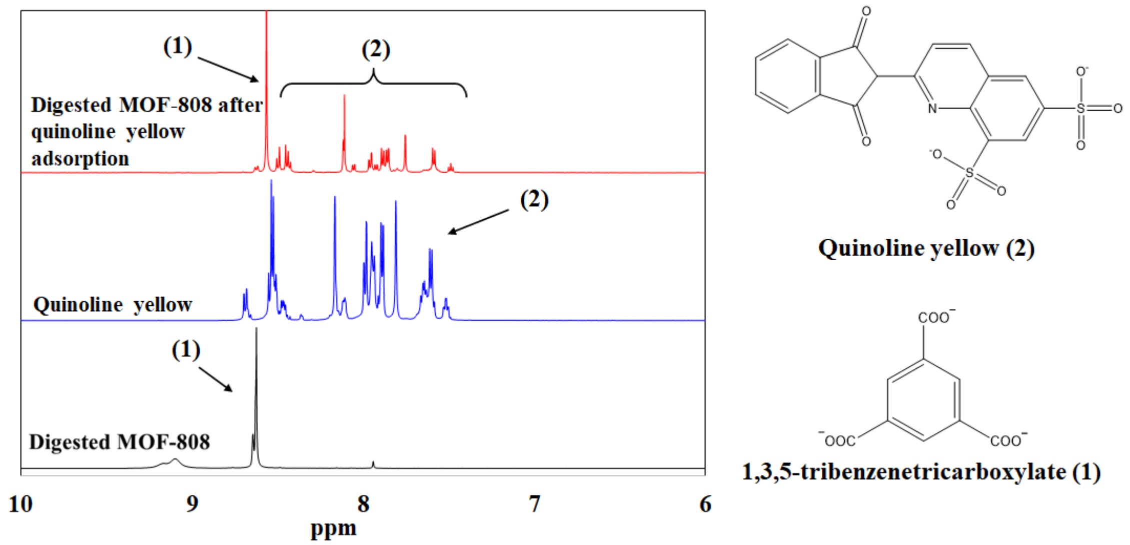

Figure S8. ^{1}H NMR spectra of digested MOF-808 after quinoline yellow adsorption.

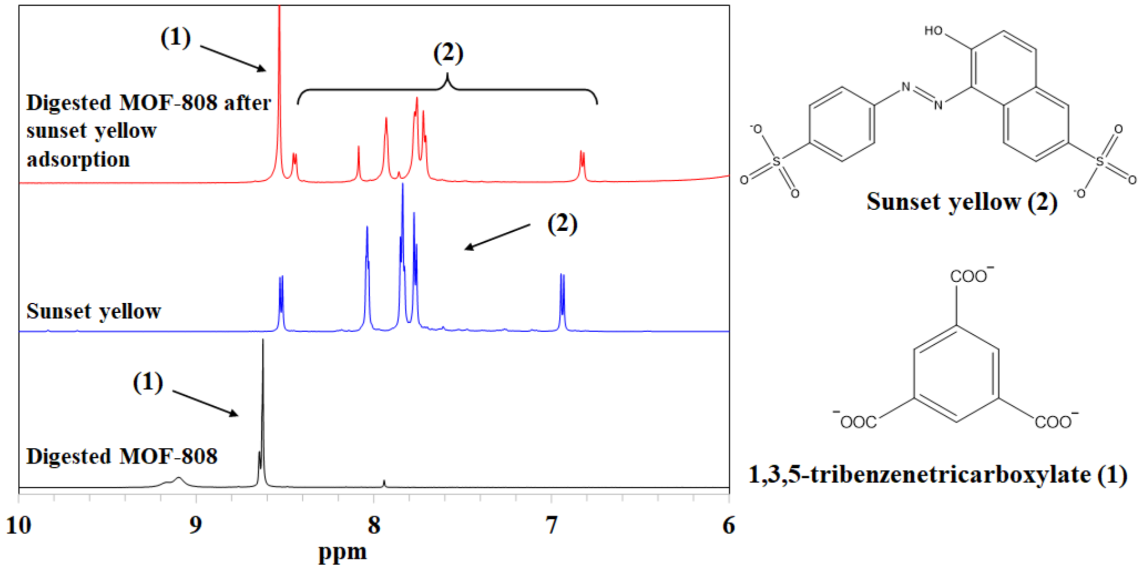

Figure S9. ^{1}H NMR spectra of digested MOF-808 after sunset yellow adsorption.

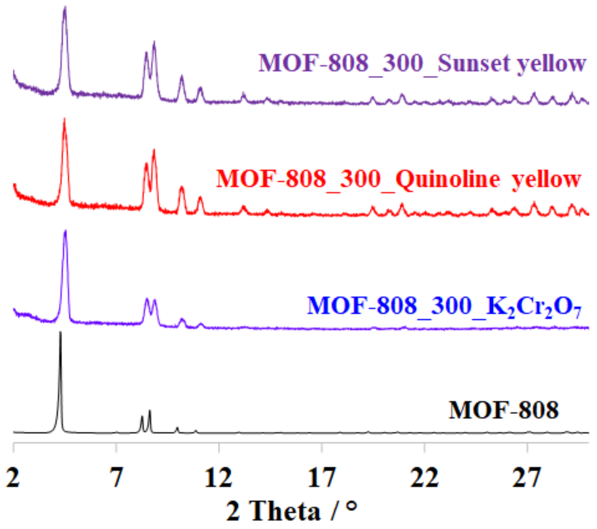

Figure S10. PXRD results of MOF-808 after employing to remove $K_2Cr_2O_7$ (blue), quinoline yellow (red), and sunset yellow (purple).

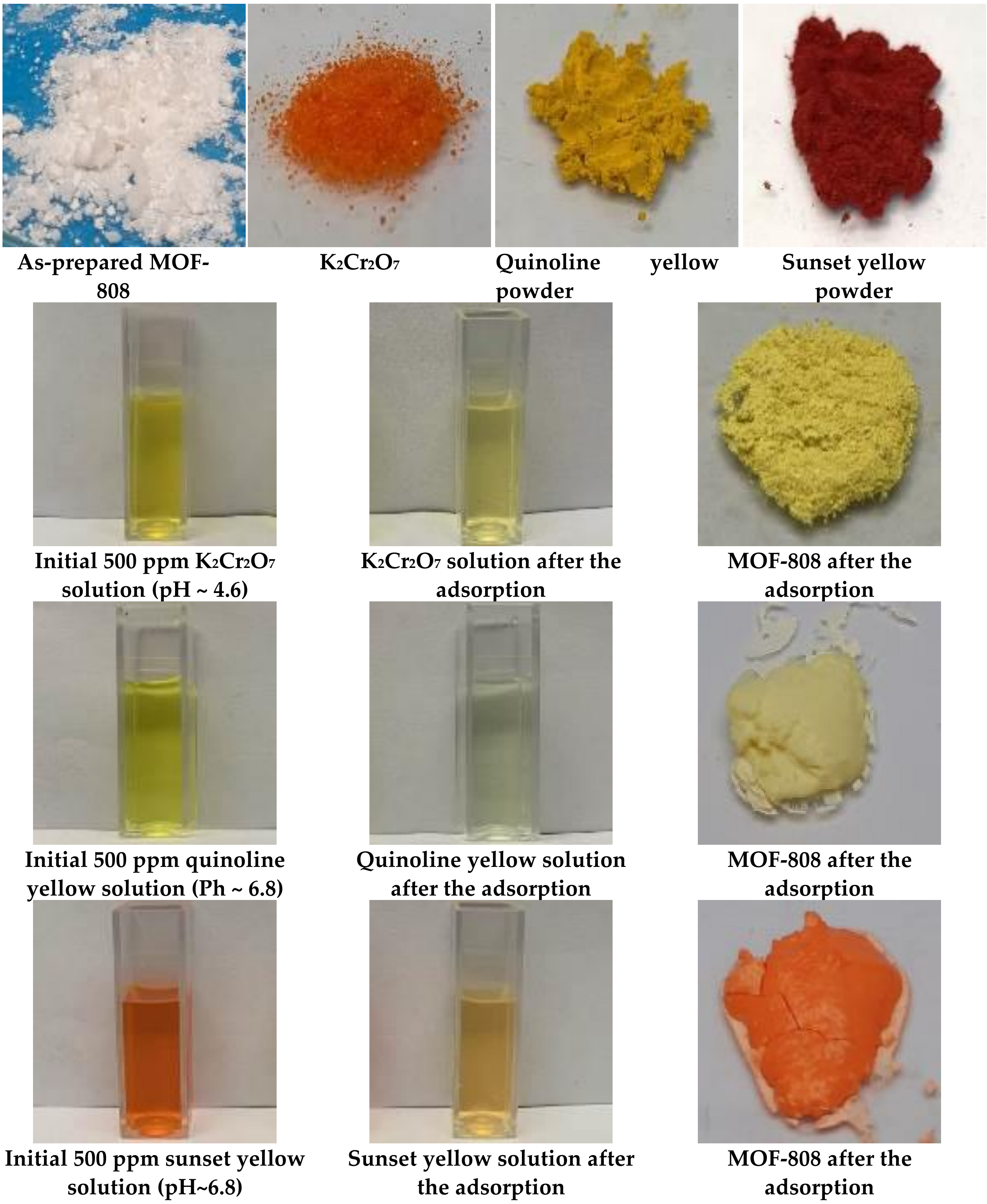

Figure S11. Photographs of the materials, liquid, and solid samples before and after the adsorption.
